# Multivessel Spontaneous Coronary Artery Dissection in an Unlikely Patient

**DOI:** 10.1155/2015/167243

**Published:** 2015-04-06

**Authors:** Waqas Jehangir, Tarek Aly, Kebir H. Bedran, Abdalla Yousif, Mark L. Niemiera

**Affiliations:** ^1^Raritan Bay Medical Center, Perth Amboy, NJ 08861, USA; ^2^Ross University School of Medicine, Portsmouth, Dominica

## Abstract

When approaching the symptom of acute onset chest pain in a previously healthy 26-year-old male, anchoring heuristic presents a challenge to healthcare workers. This diagnostic error is the healthcare professional's tendency to rely on a previous diagnosis, and, in situations where a set of symptoms might mask a rare and deadly condition, this error can prove fatal for the patient. One such condition, Spontaneous Coronary Artery Dissection (SCAD), is an uncommon and malefic presentation of coronary artery disease that can lead to myocardial infarction and sudden death. We present a case of SCAD in an otherwise healthy 26 year-old male who had been experiencing chest pain during and after sports activity. In the young, athletic male with SCAD, the danger of diagnostic error was a reality due to the broad symptomatology and the betraying demographics.

## 1. Introduction

Spontaneous Coronary Artery Dissection is a deadly and rare presentation of coronary artery disease that can lead to myocardial infarction and sudden death. As it is uncommon in young males, the recognition of SCAD in such an individual given nonspecific symptoms can mask the diagnosis at a stage where timely intervention could be lifesaving.

## 2. Case Presentation

**Figure 1:**
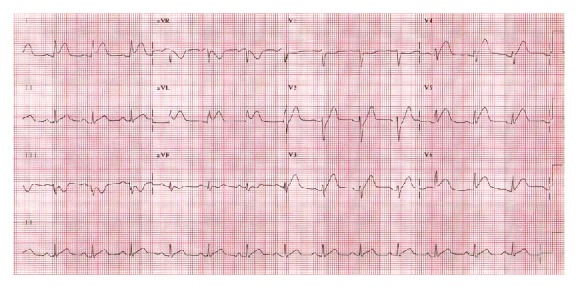
ECG showing ST elevation in the anterolateral leads.

**Figure 2:**
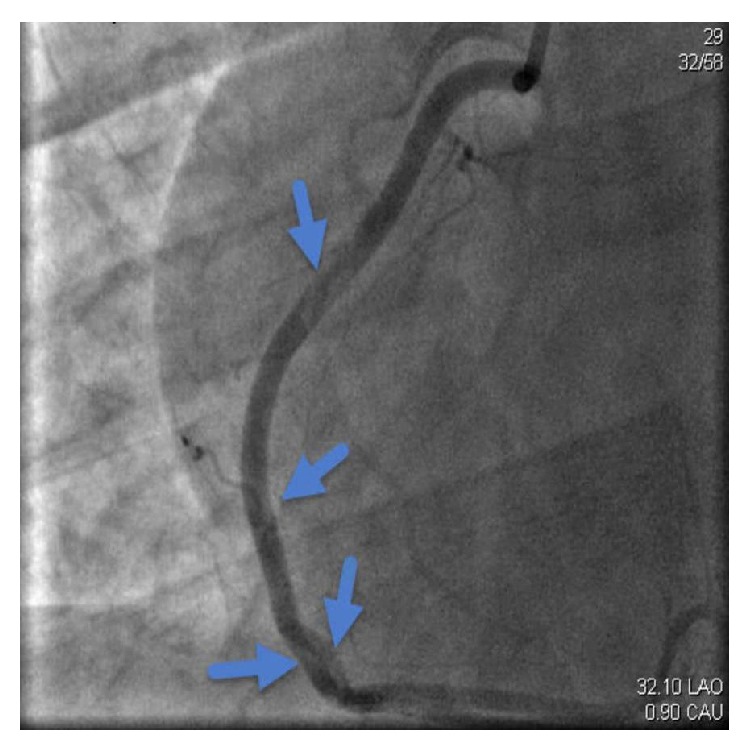
Showing 95% mid-spiral right coronary artery dissection.

**Figure 3:**
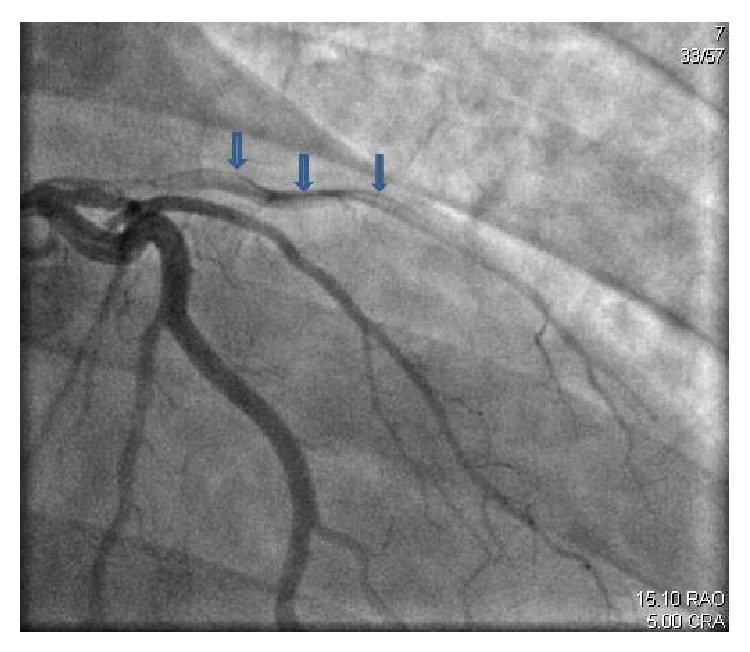
Showing total left anterior descending spontaneous dissection.

While playing cricket, a 26-year-old Indian Asian male without any significant past medical history experienced a two-week history of intermittent, retrosternal stabbing chest pain that radiated to his left arm and shoulder. The pain was pleuritic, positional, and associated with diaphoresis and nausea. The patient was diagnosed with costochondritis by his primary care physician. Seven days prior to admission, the patient had an upper respiratory tract infection with fever and chills. He denied smoking, drinking alcohol, or to using drugs; however, he reported that he had been drinking protein shakes for 3 years for muscle building. Upon physical exam, the patient was afebrile with a BP of 118/86 mmHg, PR of 111/min, and RR of 22/min. The remainder of the physical exam was completely unremarkable. Laboratory data showed Hb 15.6 g/dL, Hct 44.6, WBC 19.9 K/*μ*L, platelet count 675 K/*μ*L, glucose 157 mg/dL, BUN 21 mg/dL, Cr 1.2 mg/dL, Ca 9.7 mg/dL, albumin 4.7 g/dL, total protein 7.8 g/dL, sodium 137 mmol/L, potassium 3.3 mmol/L, chloride 98 mmol/L, and bicarbonate 20 mmol/L. Urine drug screen was negative. ECG showed ST elevation in the anterolateral leads (Figure 1). After consulting cardiology, the patient was taken to the cardiac catheterization lab for rescue angioplasty. A 95% mid-spiral right coronary artery dissection with total left anterior descending spontaneous dissection was demonstrated (Figures 2 and 3). Both the dissections were classified as type 1. The remaining vessels were normal. Left ventricular ejection fraction was measured at 15–20%. Percutaneous coronary intervention (PCI) to LAD and mid-RCA was performed. Vasculitis work-up was subsequently negative.

## 3. Discussion

Spontaneous Coronary Artery Dissection (SCAD) is a rare condition that can lead to myocardial infarction, cardiogenic shock, and sudden death. SCAD is often seen in the absence of atherosclerotic disease and is a well-recognized cause of acute coronary syndromes [1]. The disease is much more common in women than in men (58–79% female), has a mean age of 41, carries an annual incidence of 0.26 cases per 100,000, and has a prevalence of 0.07–0.28% [2]. With that stated, the presentation in a 26-year-old athletic male is a rare presentation of an already rare disease.

The pathology of SCAD entails the development of a vessel wall lesion leading to a “false lumen” and flattening of the true lumen. SCAD generally outlines an intimal-medial tear that produces a communication between the vessel lumen and intramural hematoma (the false lumen) [2]. The blood that comes into the false lumen clots and separates the detached tissue. As the blood rushes into that false lumen, the dissection occurs as the separation between the intima, media, or outer media leading to a new hollow lumen that is known as the false lumen. The false and true lumens create the typical angiographic sign of coronary dissection known as “double lumen.”

One of the dangerous aspects of SCAD involves its clinical presentation. It may present as the entire spectrum of coronary syndromes ranging from an asymptomatic, stable angina, NSTEMI, or STEMI to sudden cardiac death with 91% of patients complaining of chest discomfort [2]. In addition, almost all SCAD patients have normal blood cell counts, hemoglobin, coagulation, acute phase reactants, antibodies, and hormones. To make its ambiguity an even more challenging problem, SCAD is mostly an incidental finding during coronary angiography following STEMI or NSTEMI [2]. It is valuable to note that our patient only reported a stabbing pleuritic chest pain with left arm radiation, nausea, and diaphoresis encompassing the symptomatology of coronary syndromes in general.

SCAD has associations with atherosclerotic coronary artery disease, aortic dissection, blunt force trauma, cystic medial necrosis, and the Ehlers-Danlos syndrome; however some cases are idiopathic [3]. An association between SCAD and fibromuscular dysplasia, Marfan's syndrome, Kawasaki's disease, alpha-1 antitrypsin deficiency, and diabetic ketoacidosis has also been shown in recent studies [4]. While there are multiple connections between SCAD and the conditions listed, our patient had no pertinent medical history and thus falls into the idiopathic SCAD category.

An important facet in this case is the initial diagnosis of costochondritis made by the patient's primary care provider. The anchoring heuristic is an original diagnostic formulation that tends to influence future judgments and affects adjustments made to therapeutic plan [5]. It is of the utmost importance always not to “anchor” by demographics on the likelihood for an event to occur. Despite demographics and a decreased likelihood in a young individual, chest pain should always be taken seriously and ACS/MI should be excluded.
